# Pharmacotherapy for borderline personality disorder: A review

**DOI:** 10.1192/j.eurpsy.2021.1172

**Published:** 2021-08-13

**Authors:** M.D.C. Molina Lietor, I. Cuevas, M. Blanco Prieto

**Affiliations:** Psiquiatría, Hospital Universitario Príncipe de Asturias, Alcalá de Henares, Spain

**Keywords:** Borderline, pharmacotherapy, personality disorders

## Abstract

**Introduction:**

Borderline personality disorder (BPD) is characterized by instability of interpersonal relationships, self-image, and emotions, and by impulsivity. Although patients with BPD are misdiagnosed, some of them receive mental health treatment. Even if the first-line treatment of this disorder is psycotherapy, the patients with BPD may be highly symptomatic and are often prescribed multiple medications in a manner unsupported by evidence.

**Objectives:**

The aim of this study is to study the available evidence about the pharmacotherapy for borderline personality disorder.

**Methods:**

A review of the available literature about the management of borderline personality disorder and de pharmacotherapy for personality disorders was performed.

**Results:**

First-line treatment of the personality disorders is psycotherapy. The treatment plan for BPD may include individual and group therapy, medication, self-education, specialized substance use disorder treatment, partial hospitalization, or brief hospitalization during times of crises. Medications are generaly used only as adjuncts to psychotherapy and the adjunctive use of symptom targeted medications has been found to be useful. There is limited information to guide pharmacotherapy; preliminary evidence limits the practice of polypharmacy. Sympton-domain focused medication treatment is recommeded by some guidelines: cognitive-perceptual symtoms (low-dose antipsychotic drugs), impulsive-behavioral dyscontrol (mood stabilizers), affective dysregulation (mood stabilizers and low-dose antipsychotic drugs) and self-harm (omega-3 fatty acids).

**Conclusions:**

BPD cause significant distress and impariment of social, occupational and role functioning. The first-line treatment for BPD is psychotherapy; however symptom-focused, medication treatment of BPD is generally considered to be an adjunct to psychotherapy. The data support the efficacy of low dose antipsychotic drugs and mood stabilizers.

